# Human Adipose Mesenchymal Stem Cells Show More Efficient Angiogenesis Promotion on Endothelial Colony-Forming Cells than Umbilical Cord and Endometrium

**DOI:** 10.1155/2018/7537589

**Published:** 2018-12-13

**Authors:** Haiyuan Lu, Fan Wang, Hua Mei, Siqi Wang, Lamei Cheng

**Affiliations:** ^1^Institute of Reproduction and Stem Cell Engineering, School of Basic Medical Science, Central South University, Changsha 410078, China; ^2^Clinical Laboratory, Xiangya Hospital, Central South University, Changsha 410008, China; ^3^National Center of Human Stem Cell Research and Engineering, Changsha 410000, China

## Abstract

Angiogenesis is a complicated process in which perivascular cells play important roles. Multipotent mesenchymal stem/stromal cells (MSCs) from distinct tissues have been proved to be proangiogenic and share functional properties and gene expression profiles with perivascular cells. However, different tissues derived MSCs may exhibit different potential for clinical applications. Accordingly, comparative studies on different MSCs are essential. Here, we characterized MSCs from adipose (ADSCs), umbilical cord (UCMSCs), and endometrium (EMSCs) in terms of the surface antigen expression, differentiation ability, and the ability of angiogenesis promotion on endothelial colony-forming cells (ECFCs) both in vitro and in vivo. No significant differences in immunophenotype and differentiation were observed. In addition, three types of MSCs all located around tubular-like structures formed by ECFCs in coculture system on matrigel. But ECFCs seeded on ADSCs monolayer formed more organized capillary-like network than that on UCMSCs or EMSCs. When suspended with ECFCs in matrigel and implanted into nude mice, ADSCs promoted more functional vessel formation after 7 days. Moreover, in murine hindlimb ischemia model, cotransplantation of ECFCs with ADSCs was significantly superior to UCMSCs and EMSCs in promoting perfusion recovery and limb salvage. Furthermore, ADSC-conditioned medium (CM) contained more proangiogenic factors (such as vascular endothelial growth factor-A, platelet-derived growth factor BB, and basic fibroblast growth factor) and less inhibitory factor (such as thrombospondin-1), when compared with UCMSC-CM and EMSC-CM. And ADSC-CM more durably stabilized the vascular-like structures formed by ECFCs on matrigel and promoted ECFCs migration more efficiently. In summary, MSCs from adipose show significantly efficient promotion on angiogenesis both in vitro and in vivo than UCMSCs and EMSCs. Hence, ADSCs may be recommended as a more suitable source for treating hindlimb ischemia.

## 1. Introduction

Cell therapy has emerged as a promising strategy for treating ischemic diseases, including peripheral artery disease, myocardial infarction, stroke, and limb ischemia [1]. Human umbilical cord blood- (UCB-) derived endothelial colony-forming cells (ECFCs) [2] have been thought as an attractive cell source for ischemia therapy [3, 4] because of their ability to proliferate, differentiate into mature endothelial cells (ECs), and secret cytokines [2, 5]. However, the vessels newly formed by single ECFCs were limited in frequency and size due to the absence of the assistant cell types, such as perivascular cells [6].

Multipotent mesenchymal stem/stromal cells (MSCs), which have been demonstrated to share functional properties and gene-expression profiles with perivascular cells [7], are proved able to home to the ischemic tissue and play the perivascular role to promote blood vessel formation [8, 9]. As the originally harvested MSCs, bone marrow-derived MSCs (BMSCs) have the ability to support angiogenesis when used clinically and in various preclinical model systems [1]. However, its invasive isolation procedure, in addition with the significant decrease in relative number of MSCs and their differentiation potential with age [10], seriously limits the massive clinical application.

The emergence of alternative sources of MSCs offers more possibilities. Adipose-derived stem cells (ADSCs) are easily isolated from discarded liposuction tissue and can be maintained in vitro for extended periods of time with stable population doubling and low levels of senescence [11]. Additionally, ADSCs have the potential to augment neovascularization and improve functional recovery after ischemia [12, 13]. Umbilical cord mesenchymal stem/stromal cells (UCMSCs), which can be easily collected without invasiveness and abundantly available [14], have low expression of class I and II major histocompatibility complex, and this makes it another candidate for clinical application [15]. UCMSCs could also enhance ischemia limb perfusion [16]. Human endometrium is a highly proliferative and continuously regenerating tissue. Therefore, endometrium mesenchymal stem/stromal cells (EMSCs) involved in endometrium regeneration are thought to be a powerful tissue repair candidate. It has been confirmed that these cells produced proangiogenic factors such as MMP3, MMP10, GM-CSF, angiopoietin-2, and PDGF-BB [17]. Additionally, the injection of EMSCs in animal model of hindlimb ischemia led to the decreased limb necrosis [18]. However, MSCs isolated from various tissues might exhibit different potential for clinical applications according to their origin, and it remains unclear whether there are differences among these MSCs in angiogenesis promotion.

In this study, we cultured and identified ADSCs, UCMSCs, EMSCs, and ECFCs. Coculture system and matrigel-based subcutaneous injection of MSCs and ECFCs were used to compare the proangiogenic potential of different tissue-derived MSCs in vitro and in vivo. The cotransplantation of ECFCs with MSCs was used to evaluate the therapeutic potential in the model of hindlimb ischemia. Furthermore, we analyzed the angiogenic-related factors in MSC-conditioned medium (CM) by protein array and the effect of MSC-CM on angiogenesis-related ECFC behavior, including migration, proliferation, and stabilization. We found that ADSCs secreted more proangiogenic factors and showed stronger ability to promote vascular cell function and new blood vessel formation compared to UCMSCs and EMSCs.

## 2. Methods

### 2.1. Ethics Statement

All methods used in this study were carried out in accordance with the approved ethical guidelines of Central South University. All applicable international, national, and/or institutional guidelines for the care and use of animals were followed. The study protocol was approved by the Central South University Institutional Review Board. Informed consent was obtained from all subjects prior to the study.

### 2.2. Isolation of ECFCs

UCB from normal full-term deliveries was obtained with informed consent of the mothers from the Women and Child Health Hospital of Hunan Province. UCB-ECFCs were isolated and cultured as previously described [2]. Briefly, UCB was diluted with Dulbecco's phosphate-buffered saline (DPBS) at 1 : 1 and overlaid onto 1.077 g/ml Ficoll-Paque™ PREMIUM (GE Healthcare, USA), followed by centrifugation for 30 min at 400 g. UCB-monocytes were collected and seeded into tissue culture plates coated with fibronectin (Millipore, USA) in EGM-2 (Lonza, USA) at 37°C 5% CO_2_. Culture medium was changed every 2 days. Typical colonies appear between day 5 and 10 and were passaged at subconfluence.

### 2.3. Isolation of MSCs

Human adipose tissues were obtained by simple liposuction from the abdominal subcutaneous of three healthy female donors aged 20 to 40 (Xiangya Hospital of Central South University, Changsha, China). Adipose tissues were digested using a digestion solution containing 2 mg/mL collagenase I, 2 U/mL dispase, and 2 mg/mL hyaluronidase (all purchased from Sigma-Aldrich, MO, USA) for 90 min at 37°C. The digested tissues were centrifuged (1000 rpm for 10 min), and the stromal vascular fraction (SVF) was washed with DPBS. SVF was cultured in Dulbecco's modified Eagle's medium-F12 (DMEM/F-12) containing 10 ng/ml basic fibroblast growth factor (bFGF, GIBCO, USA) and 10% fetal bovine serum (FBS). The medium was changed every other day until the cells reach 80%–90% confluence. And cells can be harvested for expansion and freezing.

Umbilical cords (UC) were obtained from healthy infants under aseptic conditions (The Third Xiangya Hospital of Central South University) and were processed within 24 h. After the removal of blood vessels, the tissue was minced in 0.5 cm^3^ large pieces and digested with the digestion solution mentioned above for 16 h at 37°C. Cells were cultured in DMEM/F-12 containing 10 ng/ml bFGF and 10% FBS. After 3 days, nonadherent cells were removed. The medium was changed every other day. Cells can be harvested for expansion and freezing until the cells reach 80%–90% confluence.

Normal endometrial tissue was collected from women (aged 20 to 35) undergoing surgery for minor gynecologic procedures with an endometrial suction curette (Ethics Committee of Reproductive & Genetic Hospital of CITIC-XIANGYA). The tissue was washed thoroughly with PBS to remove blood, minced finely with scalpels, and digested with the digestion solution mentioned above for 2 h at 37°C. Cells were cultured in DMEM/F-12 containing 10 ng/ml bFGF and 10% FBS. After 36 h, unbounded cells were removed. The medium was changed every other day. Cells can be harvested for expansion and freezing.

### 2.4. Flow Cytometry Analysis

ECFC single-cell suspension was generated by detaching cells with TrypLE™ Express Enzyme (Gibco, USA) and resuspended to a concentration of 1 × 10^7^ cells/ml. Samples were incubated, respectively, with anti-human CD31-FITC (eBioscience, USA), VEGFR2/KDR-PE (R&D, USA), CD144-FITC (Abcam, UK), CD34-PE (Biolegend, USA), CD45-FITC (Biolegend, USA), and CD14-FITC (eBioscience, USA). MSCs were resuspended to a concentration of 1 × 10^6^ cells/ml and incubated, respectively, with anti-human CD29-PE (Biolegend, USA), CD90-PE (Biolegend, USA), CD14-FITC (Biolegend, USA), CD19-PE (Biolegend, USA), CD73-FITC (Biolegend, USA), CD105-FITC (Biolegend, USA), HLA-DR-PE (Biolegend, USA), CD34-PE (Biolegend, USA), CD45-FITC (Biolegend, USA), and CD31-FITC (eBiosciences, USA). 5 *μ*l antibody solution was added into 100 *μ*l cell suspension and incubated for 30 minutes at 4°C in the dark; 400 *μ*l of PBS was added and cells were analyzed with FACSAria I (Becton Dickinson, USA) or Accuri C6 (Becton Dickinson, USA) and Becton Dickinson CELLQuest software.

### 2.5. Adipogenesis

Adipogenic differentiation of MSCs at passage 4 was induced by the adipogenic differentiation medium consisting of DMEM/F-12 supplemented with 10% FBS, 1 mmol/L dexamethasone (Sigma-Aldrich), 0.5 mmol/L isobutylmethylxanthine (Sigma-Aldrich), 10 mg/mL recombinant human insulin (Sigma-Aldrich), and 100 mmol/L indomethacin (Guangdong Huanan Pharmaceutical Group Co. Ltd., Guangdong, China). After induction for 21 days, cells were stained with oil red O (Sigma-Aldrich) to detect the formation of neutral lipid vacuoles in adipocytes.

### 2.6. Osteogenesis

Osteogenic differentiation of MSCs was induced by the osteogenic differentiation medium consisting of DMEM/F-12 containing 10% FBS, 10 mmol/L *β*-glycerophosphate (Sigma-Aldrich), 50 *μ*M ascorbate (Sigma-Aldrich), and 100 nmol/L dexamethasone (Sigma-Aldrich). Osteogenesis was assessed by alkaline phosphatase staining (Invitrogen, CA, USA) 21 days after induction of osteogenesis.

### 2.7. Chondrogenesis

Chondrogenesis differentiation of MSCs at passage 4 was induced by the chondrocyte differentiation basal medium (Gibco, USA) after seeded at 1.6 × 10^5^ in 10 *μ*l in the well center of the 24-well cell culture plate, with medium change every 3 days. After culture for 21 days, cells were fixed with 4% paraformaldehyde and stained with 1% Alcian Blue (Gibco) to detect the synthesis of proteoglycans by chondrocytes.

### 2.8. Angiogenesis Coculture Model of Tubulogenesis on Matrigel

The coculture system on matrigel (BD, USA) was performed in 96-well plates. ECFCs and MSCs were, respectively, labeled with UEA-I (Vector Lab, USA) and CFSE (Dojindo lab, Japan). Briefly, cells suspension was washed with DPBS for 3 times and was then resuspended with DPBS at 1 × 10^6^/ml. Add 50 *μ*l UEA-I or CFSE every milliliter cell suspension. Cells were then incubated for 15 min (UEA-I labeling) or 30 min (CFSE labeling) at 37°C in the dark. Wash cells with DPBS for 3 times for the following experiments. Mixture of cells (ECFC: MSC = 2 : 3, total 1.2 × 10^4^ cells) was suspended in 50 *μ*l EGM-2, seeded on matrigel, and incubated at 37°C. Images of tubules were captured after 2 h using fluorescence camera (NIKON TE2000-U, Japan).

### 2.9. Tubule Formation Assay

MSCs were, respectively, seeded onto 24-well plates at 1.5 × 10^4^ cells/cm^2^ and incubated in L-DMEM containing 10% FBS until 80% confluency. ECFCs were seeded on the MSCs monolayer at 1 × 10^4^ cells/cm^2^ and incubated in EGM-2 medium. After 6 days coculture, UEA-I staining was performed. The images were taken using fluorescence microscope (Biotek, ELX800, USA) and Nikon photographic system (Nikon eclipse Ti-S, Japan). The quantification analysis was dealt with ImageJ software (National Institutes of Health, Bethesda, MD, USA).

### 2.10. Preparation of Conditioned Medium

MSCs at passage 4 (1 × 10^6^) were cultured in the medium mentioned above to get 80% confluency. Cells were washed with DPBS and then cultured in EBM-2 medium containing 0.2% FBS for 72 h. The medium was collected and centrifuged, and the supernatants were frozen at −70°C for use.

### 2.11. MSC-CM Mediated ECFC Angiogenesis on Matrigel

ECFCs were, respectively, suspended in 50 *μ*l EBM-2, ADSC-CM, UCMSC-CM, or EMSC-CM and seeded at 1.2 × 10^4^ cells/well on matrigel in 96-well plates at 37°C. Images of tubules were captured by the camera (NIKON Coolpix 4500, Japan) at different time intervals. The integrated vascular rings of every field of vision were counted for the following analysis.

### 2.12. ECFC Proliferation Assay

ECFCs were seeded at 2 × 10^3^ cells/well in 96-well plates for 24 h incubation. EGM-2 was changed by MSC-CM for another 24 h incubation. 5 mg/ml MTT was then pipetted into the well and incubated for 4 h in the dark. After replacing the MTT solution with 100 *μ*l DMSO per well and vibrating for 10 min, the absorbance was detected at 570 nm using Bio-Rad Model 680 96-well plate reader (Bio-Rad Laboratories Inc., Hemel Hempstead, UK).

### 2.13. ECFC Migration Assay

ECFCs were plated at 1 × 10^5^ cells/well in 1 ml EGM-2 in 24-well plates and incubated for 24 h. Scratch wounds were generated across each well using a 200 *μ*l pipette tip. The medium was replaced with MSC-CM, and the wound size was recorded by the camera (0 h). Each well was photographed again after 24 h culture. The quantification was implemented by ImageJ software (National Institutes of Health, USA).

### 2.14. Human Protein Array

According to the sample preparation instructions of RayBiotech Company, MSC-CM was prepared as mentioned above. The cytokine expression was analyzed by human protein array by RayBiotech Company (GA, USA).

### 2.15. In Vivo Vasculogenic Assay

Male BALB/c nude mice were purchased from SLAC Co., Ltd. (Shanghai, China). MSCs were mixed with ECFCs (ratio 3 : 2, total 2 × 10^6^ cells) in 200 *μ*l Matrigel (BD, USA) and the mixture was injected into the dorsal subcutaneous of 6-week-old male BALB/c nude mice. Implants were harvested after 7 days for tissue analysis. The experiment was replicated for 3 times, independently.

### 2.16. H&E Staining and Immunohistochemistry

Implants harvested from the dorsal subcutaneous of mice were fixed in 10% buffered formalin, dehydrated in 30% sucrose solution, and embedded in paraffin. Sections (7 *μ*m) were cut, mounted on slides, and stained with hematoxylin and eosin. Anti-human CD31 (Abcam, UK; 1 : 250) and anti-human alpha-smooth muscle actin (*α*-SMA; Abcam, UK; 1 : 100) were immunohistochemically stained on unstained serial sections. Negative controls using the secondary antibody alone were generated in parallel to ensure that nonspecific staining did not occur.

### 2.17. Establishment of Mouse Hindlimb Ischemic Model and Cell Transplantation

55 male BALB/C nude mice of 20–25 g in weight were anesthetized with 4% chloral hydrate by intraperitoneal injection. The right femoral artery and vein were ligated and then cut out to induce critical ischemia. Twenty-four hours later, mice were then randomly divided into 5 groups and received cell transplantations by tail vein injection: saline (NS), ECFC, ECFC + ADSC, ECFC + UCMSC, and ECFC + EMSC (*n* = 11, ratio 3 : 2, total 6.25 × 10^5^ cells).

### 2.18. Laser Doppler Perfusion Imaging

Mice were anesthetized using 4% chloral hydrate and then subjected to Laser Doppler Perfusion Imager (LDPI, Moor Instruments, Devon, UK). The animal was placed on a 37°C heating pad for 2–5 min to allow acclimation to the ambient conditions before measurement. Each mouse was imaged in triplicate. Results are reported as perfusion ratio (PR) relative to the contralateral untreated hind limb.

### 2.19. Statistical Analysis

All experiments were repeated at least 3 times independently. Data were expressed as mean ± standard deviation (SD). Comparisons between groups were performed by one-way ANOVA using SPSS17.0 (SPSS Inc., USA). For animal exterior recovery study, Kruskal-Wallis H ANOVA was used. *P* < 0.05 was considered statistically significant.

## 3. Results

### 3.1. Characterization of Human UCB-Derived ECFCs

**Figure 1:**
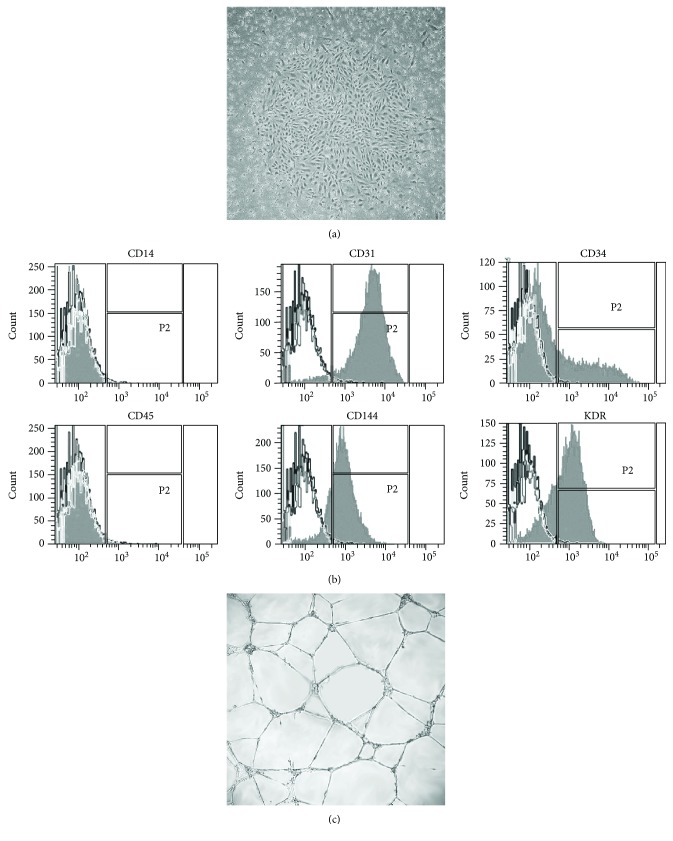
Characterization of endothelial colony-forming cells (ECFCs) isolated from umbilical cord blood. (a) Representative image displayed the cobblestone morphology of ECFCs colony (×40). Monocytes were isolated from umbilical cord blood and cultured in dishes. The cobblestone-like colony was shown after 7 days. (b) Flow cytometry analysis for CD14, CD31, CD34, CD45, CD144, and KDR in ECFCs. Black-lined histograms represent isotype-matched controls. Cells stained with fluorescent antibodies are overlaid in solid grey histograms. (c) Representative image of capillary-like structures formed by ECFCs on matrigel (×100). ECFCs were seeded on matrigel-coated culture plate, and the self-assembled capillary-like network was shown on matrigel after 2 hours.

Mononuclear cells isolated from human umbilical cord blood formed typical cobblestone-like colonies after 7–10 days culture on fibronectin-coated culture plates (Figure 1(a)). The surface markers were analyzed at passage 4. Cells were positive for endothelial marker CD31 (96.17% ± 1.03%), CD144 (VE-Cadherin, 96.77% ± 0.37%), and KDR (VEGFR2, 60.67% ± 14.03%) and were negative for the hematopoietic-related antigen CD45 (1.57% ± 0.40%) and monocyte differentiation antigen CD14 (1.23% ± 0.26%). CD34 was partly positively expressed (20.33% ± 5.89%) (Figure 1(b)). The functional characteristics of ECFCs were examined when cells were cultured on matrigel. Cells displayed interconnected capillary-like networks (Figure 1(c)).

### 3.2. Characterization of MSCs Derived from Human Adipose, Umbilical Cord, and Endometrium

**Figure 2:**
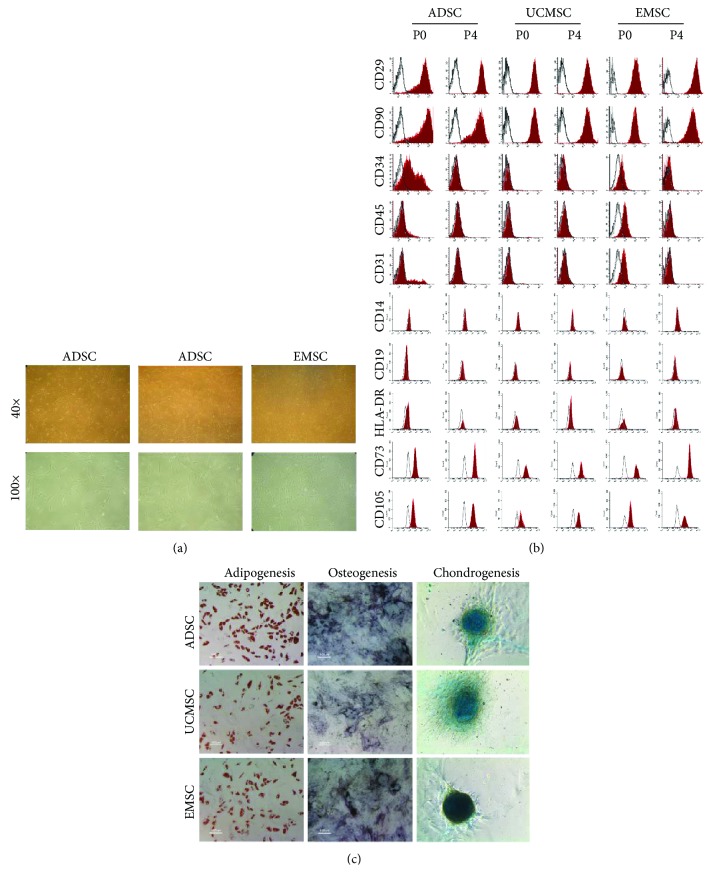
Characterization of multipotent mesenchymal stem/stromal cells (MSCs) derived from adipose, umbilical cord, and endometrium tissues. (a) Morphology of cultured adipose-derived stromal cells (ADSCs), umbilical cord mesenchymal stromal cells (UCMSCs), and endometrium mesenchymal stromal cells (EMSCs) in passage 4. Top panels and bottom panels are images at low (×40) and high (×100) magnification, respectively. All MSCs exhibited homogeneous spindle-shaped morphology. (b) Cytometric analysis of cultured MSCs in P0 and P4. Cells were labeled with antibodies against CD29, CD90, CD34, CD45, CD31, CD14, CD19, HLA-DR, CD73, and CD105 (solid red histograms). Black-lined histograms represent isotype-matched controls. (c) Multilineage differentiation potential of ADSCs, UCMSCs, and EMSCs in passage 4. Adipogenesis was assessed by staining cultures with Oil Red O to measure the accumulation of lipid vacuoles. Osteogenesis was assessed in alkaline phosphatase staining. Chondrogenesis was assessed by Alcian blue staining to detect the synthesis of proteoglycans. All MSCs showed multipotential differentiation capability.

MSCs were isolated from human adipose, umbilical cord, and endometrium tissue, respectively. Spindle-shaped morphology was observed in adherent cells at 4–7 days after initial plating, and cells at passage 4 observed in Figure 2(a) showed homogeneous fibroblastic-like morphology

We analyzed the surface markers of MSCs at passage 0 and passage 4 (Figure 2(b)). MSCs at passage 0 partly expressed the hematopoietic-related markers CD34 (ADSC, 40.60% ± 11.86%; UCMSC, 0.70% ± 0.30%; EMSC, 5.60% ± 2.10%), CD45 (ADSC, 4.3% ± 2.55%; UCMSC, 1.00% ± 0.30%; EMSC, 42.20% ± 3.52%), and endothelial marker CD31 (ADSC, 5.30% ± 4.32%; UCMSC, 0.75% ± 0.45%; EMSC, 22.80% ± 4.95%). Subsequently, all MSCs at passage 4 homogeneously showed positive expression for mesenchymal markers CD29 (ADSC, 99.53% ± 0.12%; UCMSC, 98.7%0 ± 0.30%; EMSC, 90.75% ± 0.05%), CD90 (ADSC, 97.60% ± 1.21%; UCMSC, 98.4% ± 0.33%; EMSC, 92.55% ± 0.05%), CD73 (ADSC, 98.75% ± 0.85%; UCMSC, 97.9% ± 0.5%; EMSC, 99.8% ± 0.2%), and CD105 (ADSC, 98.95% ± 0.45%; UCMSC, 93.4% ± 1.02%; EMSC, 96.95% ± 0.45%) and were negative for CD31 (<1%), CD34 (<2%), CD45 (<2%), CD14 (<1%), CD19 (<1%), and HLA-DR (<1%)

Furthermore, the adipogenic, osteogenic, and chondrogenic differentiation potential of MSCs was assessed. As shown in Figure 2(c), ADSCs, UCMSCs, and EMSCs all showed the potential differentiation to adipocytes, osteocytes, and chondrocytes.

### 3.3. ADSCs Are More Proangiogenic than UCMSCs and EMSCs In Vitro

**Figure 3:**
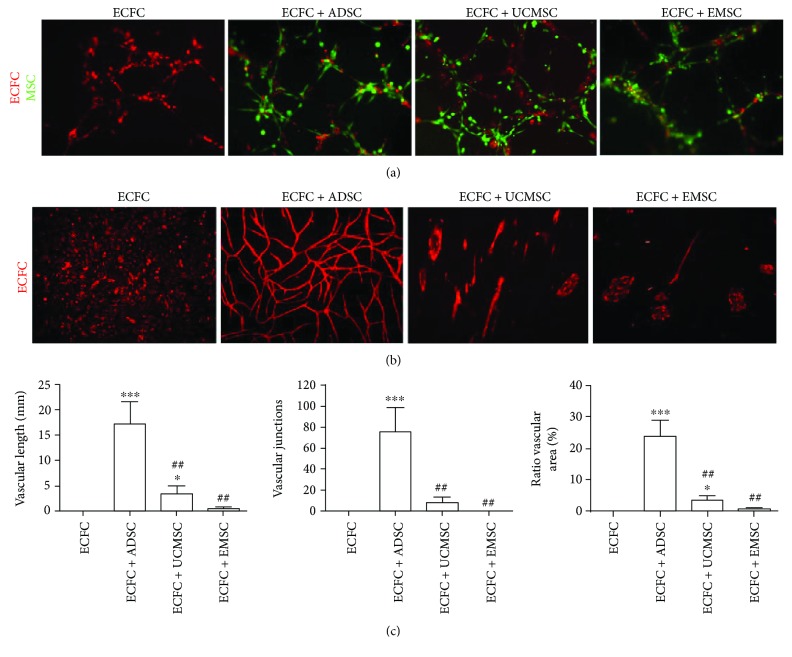
Comparison of *in vitro* proangiogenic potential of ADSCs, UCMSCs, and EMSCs. (a) Tubular-like structures formed by ECFCs at the presence of different MSCs on matrigel, respectively (×100). ECFCs and MSCs were cocultured on matrigel-coated culture plate; tubular-like networks were shown in different groups after 2 hours. ECFCs were labeled by UEA-I in red and MSCs by CFSE in green. The group of ECFC was used as control. (b) Tubular-like structures formed by ECFCs directly cultured on the monolayer of different MSCs, respectively (×40). MSCs were first seeded on culture dishes, and ECFCs were then seeded on the MSCs layers after 24 h. ECFCs were labeled by UEA-I in red. Single ECFCs were used as control. (c) Statistical analysis of vascular length, vascular junctions, and ratio of vascular area formed by ECFCs in (b). The data were determined using ImageJ software. Bars represent mean value ± SD. ^∗∗∗^ *P* < 0.001, ^∗^ *P* < 0.05, compared with ECFC group; ##*P* < 0.01, compared with ECFC + ADSC group. *N* = 3.

To compare the proangiogenic effect of three tissue-resident MSCs in vitro, coculture system of ECFCs with MSCs was performed on matrigel. As shown in Figure 3(a), ECFCs in all groups assembled into capillary-like structures. Notably, three types of MSCs all located around the tubular-like networks formed by ECFCs.

ECFCs were subsequently seeded on the monolayer of ADSCs, UCMSCs, and EMSCs, respectively. After 6 days incubation, ECFCs without feeders only showed scattered distribution with no tubular-like structure (Figure 3(b)). ECFCs seeded on ADSCs formed more organized and integrated capillary-like networks compared with UCMSCs (*P* = 0.001) and EMSCs (*P* = 0.001), and the length of vascular, the number of junctions and ratio of vascular area was 18.96 ± 3.52, 81.72 ± 14.09 and 23.97% ± 2.11% (Figures 3(b) and 3(c)), respectively. ECFCs seeded on UCMSCs only formed single tube-like structure with few junctions (length, 2.90 ± 1.31; junctions, 6.94 ± 3.22; area, 3.05% ± 1.29%), while ECFCs seeded on EMSCs only gathered together with few capillary-like structure formations (length, 0.22 ± 0.31; junctions, 0.09 ± 0.13; area, 0.40% ± 0.57%) (Figure 3(c)). Thus, ADSCs were more efficient than UCMSC and EMSC in promoting ECFCs to form tubular networks in vitro.

### 3.4. ADSCs Are More Proangiogenic than UCMSCs and EMSCs In Vivo

**Figure 4:**
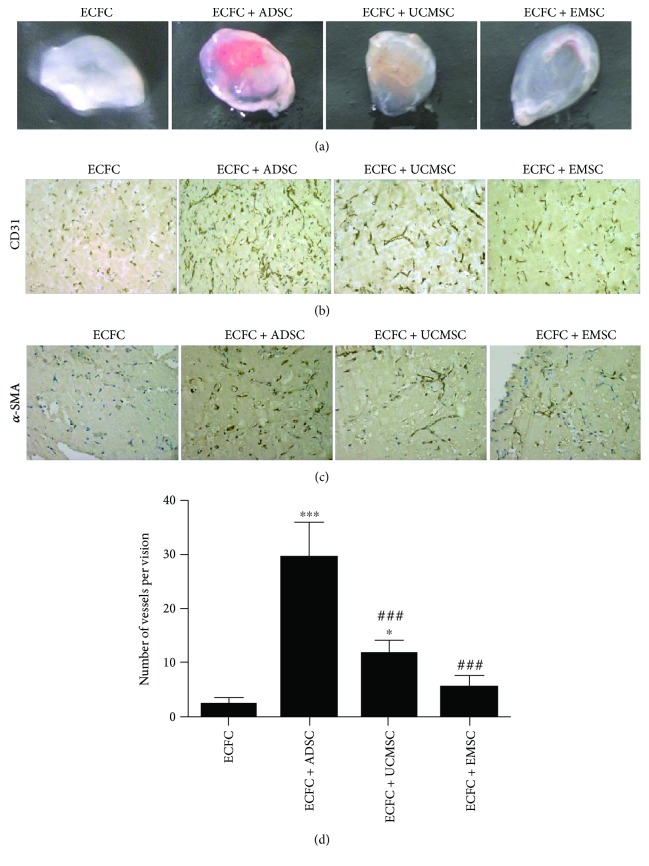
*In vivo* proangiogenic properties of ADSCs, UCMSCs, and EMSCs. (a) Macroscopic views of representative explants at day 7. Matrigel containing ECFCs with/without different MSCs was subcutaneously injected into the dorsal subcutaneous of nude mouse. The explants were excavated after 7 days. The group of ECFCs was used as control. (b) Human CD31 staining confirmed blood vessel structure and endothelial cell origin. Positive staining was shown in brown. The group of ECFCs was used as control. (c) Human *α*-SMA staining identifies human mesenchymal stromal cells within the implant. *α*-SMA positive staining was shown in brown. *α*-SMA, alpha-smooth muscle actin. (d) Statistical analysis of microvessel number in matrigel explants. Microvessel number was determined by counting the luminal structure containing erythrocytes in each field. Bars represent the mean value ± SD. ^∗∗∗^ *P* < 0.001, ^∗^ *P* < 0.05, compared with ECFC group; ###*P* < 0.001, compared with ECFC + ADSC group. *N* = 3 separate experiments.

To further assess the proangiogenic capacity of MSCs in vivo, matrigel contained ECFCs and MSCs was injected into null-mice dorsal subcutaneous. 7 days following the injection, matrigel was stripped off. As shown in Figure 4(a), the red field and shades were obviously different among different groups. The implant from the ECFC-ADSCs condition showed notably the most red. Subsequently, in order to confirm the blood vessel origin, human specific CD31 and *α*-SMA antibody were used to identify endothelial cells and stromal cells, respectively. As shown in Figures 4(b) and 4(c), vessels formed in implants were positively stained by hCD31, and positive a-SMA staining was circumferentially localized around the newly developed blood vessels. In details, vessel-like structures formed by ECFCs alone were very small. Coinjection of ADSCs and ECFCs brought evenly distributed vessel-like structures with almost uniform lumen size. But the lumen size of vessels formed in UCMSCs and EMSCs groups was quite different, or even showed a state of disorder. Meanwhile, the results of histology revealed the differences in the vessel numbers among the various groups. As shown in Figure 4(d), ECFCs alone only formed very few vessels. ADSCs and UCMSCs promoted a significant increase of vessel numbers, while no statistical difference was observed in EMSCs group compared with ECFCs alone. Thereinto, ADSCs groups formed more vessel-like structures compared with UCMSCs (*P* < 0.001) and EMSCs (*P* < 0.001). Collectively, these results indicated that ADSCs promoted the microvessel formation of ECFCs more efficiently in vivo.

### 3.5. Cotransplantation of ADSCs and ECFCs More Efficiently Promotes the Revascularization in Hindlimb Ischemia Mouse Model

**Figure 5:**
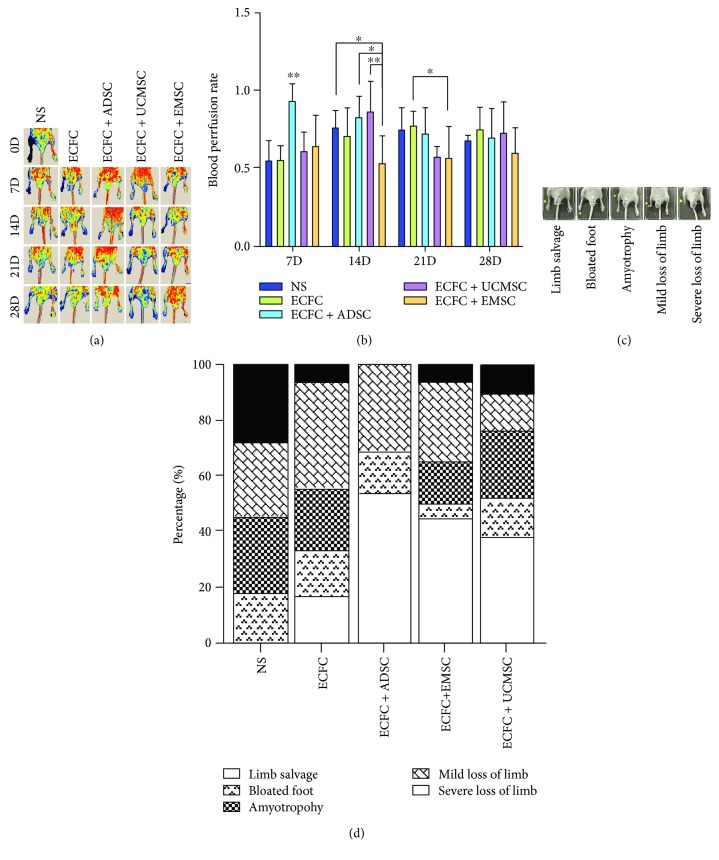
The therapeutic efficacy of different cells in murine hindlimb ischemia model. (a) Perfusion heat maps of different cell groups on day 0, 7, 14, 21, and 28, respectively. Different MSCs with ECFCs were transplanted by tail intravenous injection 24 h after right femoral and saphenous artery ligation. Laser Doppler Perfusion Imaging (LDPI) visualized the dynamic changes in hind limb perfusion of different cytotherapy groups on indicated time points. Normal saline (NS) was used as control. (b) Blood perfusion rate of different cell groups. Blood perfusion was quantified using perfusion rate, i.e., the rate of average LDPI index of ischemic limb (left) to nonischemic hind limb (right). Bars represent the mean perfusion rate ± SD. ^∗∗^ *P* < 0.01, ^∗^ *P* < 0.05. *N* = 11. NS was used as control. (c) Representative images of five progressive exterior morphological recovery levels of ischemic mice on day 28. From left to right are limb salvage, bloated foot, amyotrophy, mild loss of limb, and severe loss of limb, respectively (yellow arrow). (d) Percentage bar chart of exterior recovery statistics in different cytotherapy groups. Kruskal-Wallis H ANOVA was used to analyze the composition. NS was used as control. *N* = 11.

The proangiogenic properties of MSCs were further explored in mouse hindlimb ischemic model. After 24 h of right femoral artery ligation and excision surgery, ECFCs and MSCs were cotransplanted into mice by tail intravenous injection. The perfusion of ischemia limb was detected by LDPI at day 0, 7, 14, 21, and 28, respectively.  Figure 5(a) was the representative of the results. The results showed that cotransplantation of ECFCs with ADSCs significantly improved blood flow of ischemic limb at day 7 compared with other groups (0.94 ± 0.10; *P* = 0.001) (Figure 5(b)). At day 14, EMSC-ECFC group showed relatively lower perfusion rate (0.52 ± 0.13), while no difference was observed among other groups. The data at day 21 indicated the unified trend of blood flow recovery in different groups over time. No statistical difference was observed in blood perfusion rate among all groups until day 28.

The recovery of ischemic hindlimb on day 28 was defined as five progressive levels: limb salvage, bloated foot, amyotrophy, mild loss of limb, and severe loss of limb (Figure 5(c)). As shown in Figure 5(d), cell transplantation considerably raised the limb salvage rate and reduced the limb loss. The proportion of limb loss (both mild loss and severe loss of limb) in NS group was over 50%, and no mice showed final limb salvage; the cotransplantation of ECFCs and MSCs was significantly superior to ECFC transplantation alone. The severe loss rate was 5.6% in ECFC group, and ADSCs-ECFCs cotransplantation showed significantly the best effect for the recovery of mouse ischemic hindlimb with no severe loss of limb (*P* = 0.048). Although the blood flow of ischemic hindlimb did not show statistical differences among groups on day 28, the perfusion rate of blood flow in ADSCs group was higher than other groups on day 7. Thus, rapid revascularization of ischemic tissues is essential for the restoration of their physiological function.

### 3.6. ADSCs Secrete More Proangiogenic-Related Factors

**Figure 6:**
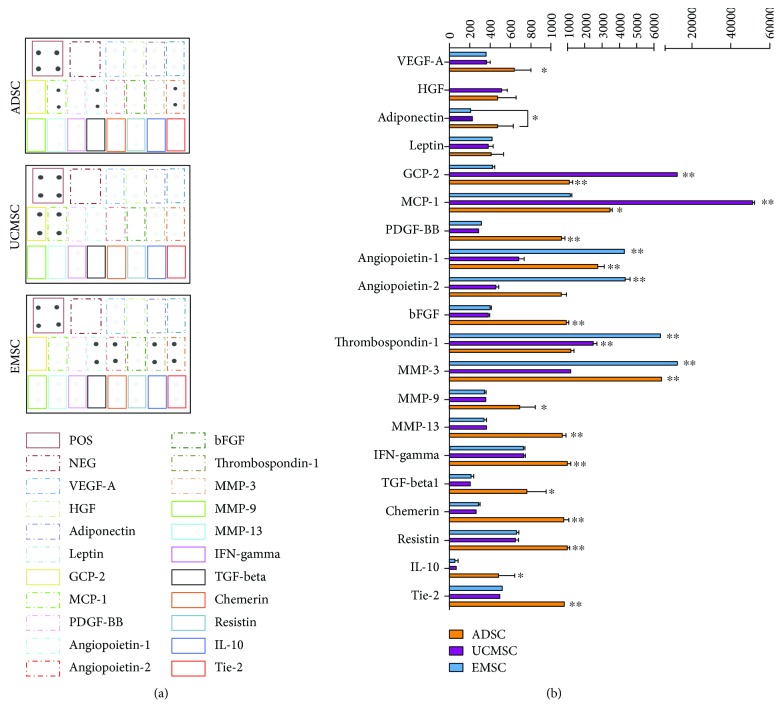
ADSC-conditioned medium (CM) is enriched in angiogenic-related factors in comparison with UCMSC-CM and EMSC-CM. (a) Human cytokine antibody array analysis showed the angiogenic proteome profiler by testing ADSC-CM, UCMSC-CM, and EMSC-CM. The position of selected cytokines in the membranes was marked in colorful boxes. Positive control (POS) and negative control (NEG) were ranked in the top left corner of each membrane. (b) Statistical analysis of the expression levels of selected cytokines in MSC-CMs was carried out by densitometry in units of pixel density adjusted for background. Bars represent the mean ± SD. ^∗^ *P* < 0.05. ^∗∗^ *P* < 0.01.

We queried whether the different promoting effects of MSCs on vessel formation of ECFCs were caused by MSC-secreted proangiogenic cytokines. The profiles of angiogenic-related cytokines in ADSC-CM, UCMSC-CM, and EMSC-CM were analyzed by cytokine antibody array. The results showed that three types of MSC-CMs all contained a variety of angiogenic cytokines (Figure 6(a)). As shown in Figure 6(b), ADSCs secreted significantly higher level of many cytokines and MMPs that directly promote endothelial cell migration, proliferation, and endothelial sprout, such as VEGF-A (*P* = 0.047, compared with UCMSC; *P* = 0.049, compared with EMSC), bFGF (*P* = 0.001, UCMSC; *P* = 0.002, EMSC), PDGF-BB (*P* = 0.007, UCMSC; *P* = 0.009, EMSC), TGF-*β*1 (*P* = 0.013, UCMSC; *P* = 0.014, EMSC), IFN-gamma (*P* = 0.005, UCMSC; *P* = 0.005, EMSC), IL-10 (*P* = 0.018, UCMSC; *P* = 0.016, EMSC), chemerin (*P* = 0.007, UCMSC; *P* = 0.008, EMSC), MMP-9 (*P* = 0.03, UCMSC; *P* = 0.027, EMSC), and MMP-13 (*P* = 0.008, UCMSC; *P* = 0.007, EMSC). UCMSC secreted more chemotactic factors MCP-1 (*P* = 0.001) and GCP-2 (*P* = 0.001) compared with ADSC and EMSC. EMSC showed predominance in the expression of MMP-3 (*P* = 0.001) and angiopoietin-1/2 (*P* = 0.001). However, ADSC secreted less thrombospondin-1, a powerful inhibitor of ECs' proliferation and migration, compared to UCMSC (*P* = 0.009) or EMSC (*P* = 0.001). Collectively, ADSCs secreted more proangiogenic cytokines and less inhibitor in comparison to UCMSCs and EMSCs.

### 3.7. ADSC-CM Promote Vessel-like Structure More Stabilization

**Figure 7:**
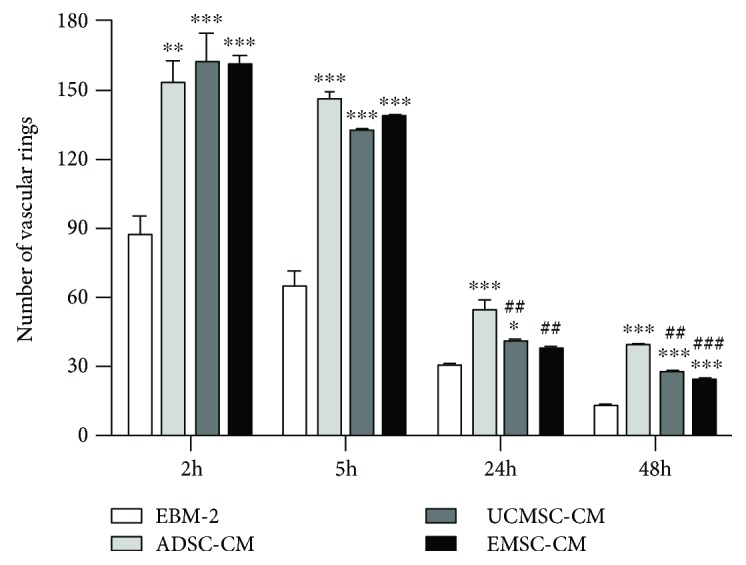
MSC-CMs stabilize the capillary-like structures formed by ECFCs on matrigel. Statistical analysis of vascular rings number in MSC-CMs was carried out at different time points. Bars represent the mean value ± SD. ^∗^ *P* < 0.05; ^∗∗^ *P* < 0.01; ^∗∗∗^ *P* < 0.001; compared with EBM-2. ##*P* < 0.01; ###*P* < 0.001; compared with ADSC-CM.

ECFCs seeded on matrigel were cultured in EBM-2 medium containing MSC-CMs. The capillary-like structures were counted at 2 h, 5 h, 24 h, and 48 h, respectively (Supplementary Figure S1). ECFCs in all conditions formed interconnected capillary-like networks at 2 h, and the number of capillary-like rings showed no differences among different CM groups (Figure 7). But the addition of MSC-CMs significantly increased the formation of capillary-like networks (EBM-2, 87.5 ± 5.5; ADSC-CM, 153 ± 7, *P* = 0.002; UCMSC-CM, 162 ± 9, *P* < 0.001; EMSC-CM, 161 ± 3, *P* < 0.001; compared with EBM-2). The networks were gradually collapsed over time. The number of the remaining vascular rings in ADSC-CM (24 h, 55 ± 3; 48 h, 39.5 ± 0.5) was significantly more than that in UCMSC-CM (24 h, 41.5 ± 0.5, *P* = 0.005; 48 h, 27.5 ± 1.5, *P* = 0.003) and EMSC-CM (24 h, 37 ± 1, *P* = 0.002; 48 h, 23.5 ± 1.5, *P* < 0.001) (Online Resource 1). Thus, ADSC-CM was more efficient than UCMSC-CM and EMSC-CM in maintaining the stability of endothelial networks on matrigel.

### 3.8. ADSC-CM Significantly Increases ECFC Migration

**Figure 8:**
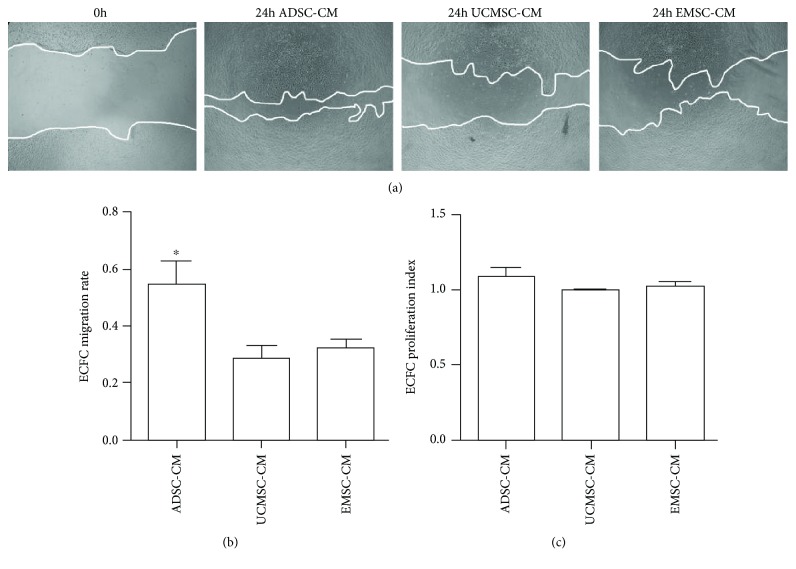
Effect of MSC-CMs on migration and proliferation of ECFCs. (a) Representative images of ECFCs wound healing assay in different MSC-CMs (×40). ECFCs monolayer was scratched on 0 h, and the medium was replaced by ADSC-CM, UCMSC-CM, and EMSC-CM, respectively. EBM-2 was used as control. Images of scratch area were captured after 24 h. (b) The quantification of ECFCs migration ratio in different mediums. The ratio of migration was defined as follows: *(blank area on 0 h – blank area on 24 h)/blank area on 0 h.* Bars represent mean ± SD. ^∗^ *P* < 0.05. *N* = 3. (c) The quantification of ECFCs proliferation index in different mediums. Proliferation index was quantified by MTT measurement. Bars represent mean ± SD. ^∗^ *P* < 0.05. *N* = 3.

Subsequently, we analyzed the effects of MSC-CM on ECFC migration and proliferation in vitro. As shown in Figure 8(a), ADSC-CM showed better effect on promoting ECFC migration compared with UCMSC-CM (*P* = 0.018) and EMSC-CM (*P* = 0.01). The migration rate of ECFC was, respectively, 54.87% ± 11.83% (in ADSC-CM), 28.40% ± 6.37% (in UCMSC-CM), 32.24% ± 4.51% (in EMSC-CM), and 27.10% ± 7.21% (in EBM-2)(Figure 8(b)). No significant difference in ECFC proliferation was observed among groups (Figure 8(c)).

## 4. Discussion

Previously, transplantation of ECFC is found to promote the neovascularization of ischemic tissues [19]. But the vascular-like structures formed by only ECs show unstable, which may be related to the lack of perivascular cell types [6]. MSCs not only show similar characterization with perivascular cells [7] but also have close association with ECFCs. For example, factors secreted by MSCs and ECFCs are found to have complementary effects on angiogenesis in vitro [20]. Besides, ECFCs can regulate the regenerative potential of MSCs [21], while MSCs can secrete cytokines to promote the survival, proliferation, and migration of ECFCs [22]. Another interesting report shows that direct contact between MSC and bone marrow-derived endothelial cells expressing Flk-1+ CD34+ can induce a pericyte-like phenotype and angiogenesis in MSCs [23]. Accordingly, the combined application of ECFC and MSC is an ideal model for ischemia cytotherapy.

Notably, since MSCs have wide tissue origin, and the origin of tissue and its specific niches influence the plasticity of MSCs [24], the direct comparison among tissues is meaningful and remains problematic. Previous studies have described potent differences existing among different tissue-derived MSCs. Kern et al. compared the proliferative capacity of MSCs derived from adipose tissue, bone marrow, and umbilical cord blood and found that UCB-MSCs could be cultured longest and showed the highest proliferation capacity, whereas BMSCs possessed the shortest culture period and the lowest proliferation capacity [25]. Additionally, analysis of BMSCs and UCB-MSCs indicate the former show higher expression levels of genes associated with osteogenic differentiation, while the latter exhibit higher expression of angiogenesis related genes [25]. Jin et al. also found UCB-MSCs had the highest rate of cell proliferation, but expressed significantly lower level of senescence markers, including p53, p21, and p16 [26]. But MSCs from adipose tissue is indicated to show a better migration and homing capacity when compared to BMSCs [27]. But whether different MSCs will result in different effects is not yet conclusive. It is indicated that MSCs from placental amnion, chorion, and umbilical cord exert similar beneficial effects on wound closure and neovascularization [28], and bone marrow, adipose tissue, and dental pulp are also proved to serve as a universal MSC source for burn wound healing [29]. Conversely, Aboulhoda and Abd El Fattah demonstrated that ADSCs show statistically significant improvement in wound healing than BMSCs [30]. And adipose-derived MSCs are also proved to exhibit greater angiogenic potential in comparison with BMSCs [31] and UCMSCs [22]. These are partly in accordance with our data. We found ADSCs showed a significant advantage in both in vitro and in vivo angiogenesis promotion in comparison to UCMSC and EMSC. But Lin et al. indicated a different conclusion in a research using four murine tissues derived MSCs, including white adipose tissue, bone marrow, skeletal muscle, and myocardium [32]. The authors found all four MSCs similarly secreted a series of proangiogenic factors and generated extensive vascular network with ECFCs in vivo and concluded there were no differences among various murine tissues derived MSCs in angiogenesis promotion. This difference may be due to the different cell strain and source used in research.

We observed that all three types of MSCs are capable of locating around both the capillary-like structures formed by ECFCs on matrigel in vitro and the neovessels formed in subcutaneously implanted matrigel. These results indicated that MSCs might act as perivascular cell role to provide support to the neovessels. This is in accordance with the previous research that demonstrates MSCs can stabilize and maintain vascular structures in vivo [33], with another study indicates MSCs as tissue-specific progenitors residing in close proximity to the microvasculature of various organs [34].

In this study, the codelivery of ECFCs and MSCs resulted in different improved rate of recovery and the reduced risk of limb loss. This is in accordance with the evidence of animal studies, in which MSCs transplantation is found to form new blood vessels to restore blood flow of ischemic limbs [35, 36]. Notably, we found that treatment with ECFCs and ADSCs led to the best prognosis. It is considered as a result of its advantageous blood flow perfusion at earlier day 7, despite no observed difference after day 7. Since the extensive cell death and tissue damage can ensue during one to two weeks [37], it is essential to accelerate the neovascularization process as early as possible. Similar conclusion has been previously reported by Pedersen et al. [38] and Moon et al. [39].

Although two mechanisms about MSCs' proangiogenic property exist simultaneously according to the previous observations, the paracrine secretion is more important in comparison with direct differentiation [24]. Previous study and our finding consistently show that MSC-CMs have positive effect on angiogenesis [40] and could promote tube formation of ECs in vitro. But the secretome of different tissue-derived MSCs is dependent on cell source. Accordingly, in this study, we analyzed the angiogenic-related factors of three different tissue-derived MSC-CMs by cytokine antibody array. The factors we selected could be clarified into the following groups: growth factors (VEGF-A, TGF-beta1, bFGF, PDGF-BB, and HGF), angiopoietin and receptor (ANG1, ANG2, and TIE-2), matrix metalloproteinase (MMP-3, MMP-9, and MMP-13), chemokines (MCP-1 and GCP-2), inflammatory factors (IL-10 and IFN-gamma), angiogenesis inhibitor (Thrombospondin-1), and adipose-related factors (adiponectin, leptin, chemerin, and resistin). Our results showed that VEGF-A, TGF-beta1, bFGF, and HGF, which directly promote ECs survival, proliferation, and migration, were all expressed in ADSCs, UCMSCs, and EMSCs. But ADSCs showed the highest expression of them. Interestingly, Arutyunyan et al. detected no soluble VEGF-A in UCMSC-CM [41], which is inconsistent with our finding. As the major cytokine of pericyte recruitment in physiological angiogenesis, there is a controversy regarding the expression of PDGF-BB in MSCs. Liang et al. detected PDGF-BB expression in placenta derived MSCs [24], while Chen et al. demonstrated that PDGF-BB is not expressed in MSCs [42]. In our study, three MSCs all expressed PDGF-BB, and that in ADSCs was the highest. Moreover, angiopoietins (angiopoietin-1 and angiopoietin-2) and their receptor Tie-2 are crucial to regulate sprouting angiogenesis [43]. There were also differences in their expression in MSCs. Ang1 and Ang2 were highly expressed in EMSCs and ADSCs. As for the soluble fragment of Tie-2, it was also most highly detected in ADSC-CM. MMP-3, MMP-9, and MMP-13 are three important metalloproteinases in the degradation of ECM to promote ECs sprouting and invasion [44, 45]. The stimulation of MMP-3 is demonstrated to be dependent on heterotypic cell contacts between ECFCs and MSCs, with a significant reduction when MSC was replaced with MSC-CM [46]. We believe that MMPs secreted by MSCs are a more negligible part, and cell source might bring different expression level. Here, we detected that MMP-9 and MMP-13 were both highly expressed in ADSC-CM, while EMSC-CM showed high expression of MMP-3. This indicated that ADSCs and EMSCs might induce more ECs sprout. Factors that promote the migration of ECs are another focal point, including GCP-2, MCP-1, and HGF [47]. It is well known that MCP-1 and HGF is included in MSC secretome, but secretion of MCP-1 and HGF by UCMSCs is found significantly more intensive than BMSCs or ADSCs [48, 49]. Our research shows that expression of GCP-2, MCP-1, and HGF was higher in UCMSCs and ADSCs than in EMSCs, while GCP2 and MCP-1 expressed in UCMSCs were the highest. In addition to well-known angiogenic cytokines such as VEGF and bFGF, a growing number of evidence suggests that many inflammatory factors influence ECs migration, proliferation, and tube formation process [50, 51]. Human MSC are demonstrated to express the immunosuppressive cytokines HGF, IL-10, and TGF-beta1 [52], and we found that their expression in ADSC-CM showed predominantly higher, with only similar HGF level in UCMSC-CM. Although the proinflammatory cytokine IFN-*γ* is also the highest expressed in ADSC-CM, it is proved that IFN-γ could strengthen MSCs immunosuppression by upregulating its expression of HGF and TGF-beta1 [52]. This indicated that ADSCs could more efficiently inhibit the inflammation response, which may be beneficial for its angiogenesis promotion. Furthermore, as a powerful inhibitor of ECs' proliferation and migration, the expression of thrombospondin-1 was significantly lower in ADSCs than UCMSCs or EMSCs. All these results collectively demonstrated that ADSCs secreted more cytokines that directly or indirectly promote angiogenesis. Further study is in great need to validate the actual function of cytokine networks in the context of in vivo condition.

EC's proliferation and migration play important roles in angiogenesis. In our setting, MSC-CMs all induced the promoted migration of ECFC. Similar results have been reported previously that P-MSC, UC-MSC, and BM-MSC CM significantly promoted the migration of human umbilical vein endothelial cell (HUVEC) [24]. Besides, Arutyunyan et al. also revealed that UCMSC-CM effectively stimulates the migration of EA.hy926 cells [41]. But published evidence for MSC's stimulation on EC's proliferation is rather controversial. In our experiments, three different MSC-CMs have no promotion on ECFC's proliferation. This is supported by Steiner et al. who also indicated that CM from MSCs had no effect on T17b endothelial cell line proliferation [46]. It is also shown that bone marrow-derived MSCs have no effect of EA.hy 926 cell growth either [20]. But Arutyunyan et al. demonstrated that UCMSC-CM promotes EA.hy926 EC line's proliferation [41], which is consistent with the results reported by Choi et al. using HUVEC [15]. The controversial results may be due to the variety of cell sources and isolation methods [36].

In addition, different mouse strains were demonstrated to show different angiogenesis rate and growth factor expression [53]. So a limitation of this study was the lack of usage of different mouse strains, which may need further study in the future. Besides, the limited number of human tissue samples that we used is another limitation. Although the consistency of the results among different samples is well, a larger number of tissue samples for evaluation are still in great need.

In conclusion, this study demonstrated that MSCs from human adipose are more efficient than that from umbilical cord and endometrium tissues in both in vitro and in vivo angiogenesis promotion. This might be associated with ADSCs' secreting more proangiogenic cytokines and less inhibitor, maintaining the vascular-like structures stability more efficaciously and promoting ECFC migration more efficiently. Therefore, ADSCs may be a more ideal cell source for clinical treatment of ischemia vascular disease.

## Data Availability

The data used to support the findings of this study are included within the article and the supplementary materials.

## Supplementary Materials

Supplementary Materials Figure S1: MSC-CMs stabilize the capillary-like structures formed by ECFCs on matrigel.
