# Supplementary material for: Human Adipose Mesenchymal Stem Cells Show More Efficient Angiogenesis Promotion on Endothelial Colony-Forming Cells than Umbilical Cord and Endometrium
Source: Stem Cells Int. 2018 Dec 13;2018:7537589. doi: 10.1155/2018/7537589 (PMC6311802; doi:10.1155/2018/7537589)
Supplement: Supplementary Materials — Figure S1: MSC-CMs stabilize the capillary-like structures formed by ECFCs on matrigel. [file 7537589.f1.docx]

## Title

## Human adipose mesenchymal stem cells show more efficient angiogenesis promotion on endothelial colony forming cells than umbilical cord and endometrium

## Authors

**Haiyuan Lu^1, 3^, Fan Wang^2^, Hua Mei^2^, Siqi Wang^2^, Lamei Cheng^1, 2,^** *

1. Institute of Reproduction and Stem Cell Engineering, School of Basic Medical Science, Central South University, Changsha 410078, China

2. National Center of Human Stem Cell Research and Engineering, Changsha 410000, China

3. Clinical Laboratory, Xiangya Hospital, Central South University, Changsha 410008, China

* Corresponding to Professor Lamei Cheng. Address: National Center of Human Stem Cell Research and Engineering, No.8 Luyun Road, Changsha 410000, China; Fax: 86-731-88395618; Tel: 86-731-88395618; E-mail address: mmlamei@163.com.

**Supplementary Figure S1**

**
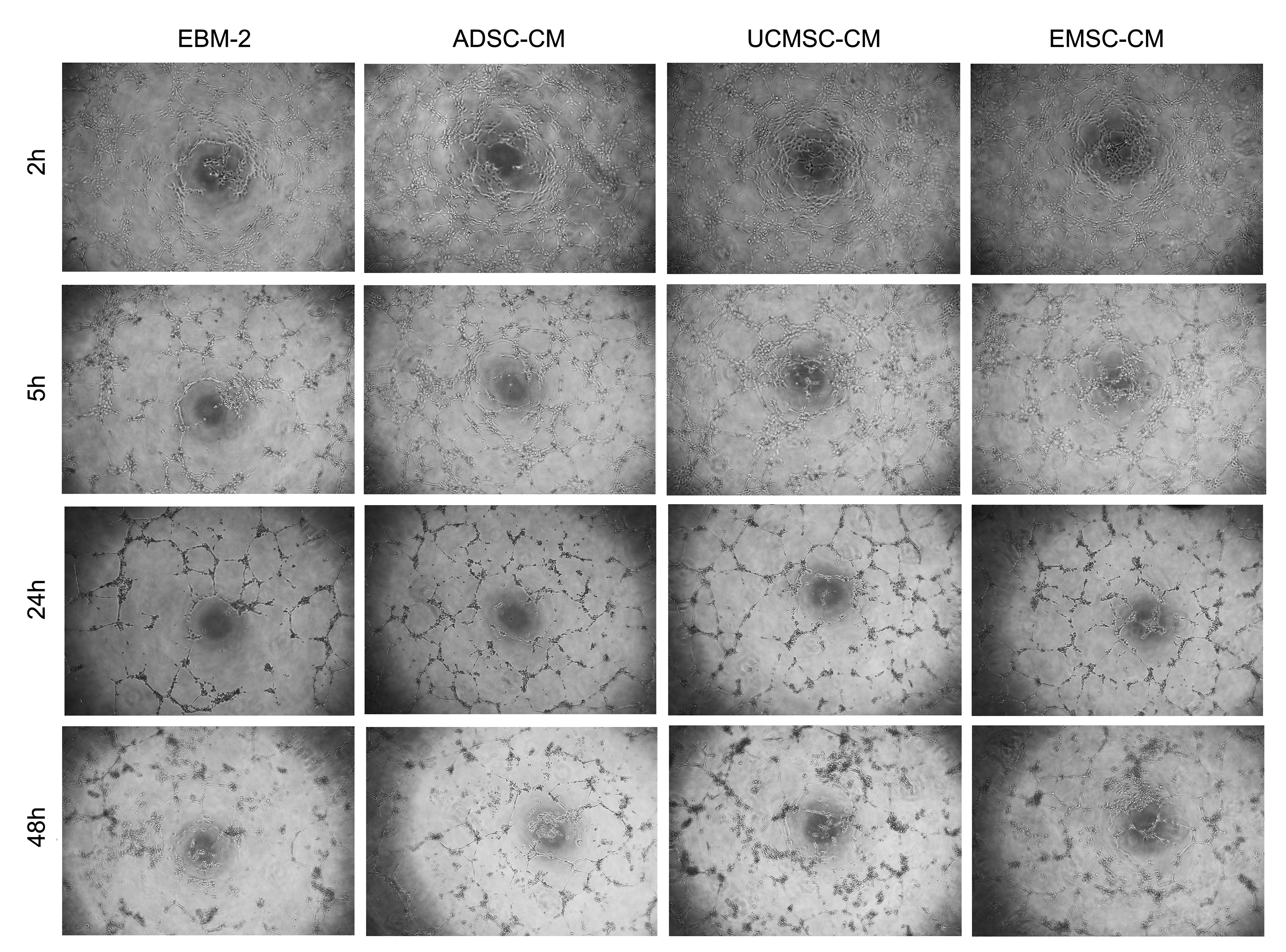
**

**Figure S1. MSC-CMs stabilize the capillary-like structures formed by ECFCs on matrigel.**

Representative images of vascular-like structures formed by ECFCs on matrigel at the presence of different MSC-CMs (×40). EBM-2 was used as control. Photographs were taken at different time points, including 2h, 5, 24h, and 48h.
